# Time to Endovascular Treatment and Clinical Outcome in Acute Ischemic Stroke With M2‐Segment Occlusion

**DOI:** 10.1161/JAHA.125.043984

**Published:** 2025-11-11

**Authors:** Sterre Dassen, Adriaan van Es, Bob Roozenbeek, Rob van de Graaf, Yvo Roos, Charles Majoie, Elyas Ghariq, Nyika Kruyt, Urs Fischer, Robert van Oostenbrugge, Wim van Zwam, Julie Staals

**Affiliations:** ^1^ School for Cardiovascular Diseases (CARIM) Maastricht University Maastricht the Netherlands; ^2^ Department of Neurology Maastricht University Medical Center Maastricht the Netherlands; ^3^ Department of Radiology Leiden University Medical Center Leiden the Netherlands; ^4^ University Neurovascular Centre (UNVC) Leiden‐The Hague Leiden the Netherlands; ^5^ Department of Neurology Erasmus MC, University Medical Center Rotterdam Rotterdam the Netherlands; ^6^ Department of Radiology and Nuclear Medicine Erasmus MC, University Medical Center Rotterdam Rotterdam the Netherlands; ^7^ Department of Neurology Amsterdam UMC location University of Amsterdam Amsterdam the Netherlands; ^8^ Department of Radiology and Nuclear Medicine Amsterdam Neurosciences, Amsterdam UMC location University of Amsterdam Amsterdam the Netherlands; ^9^ Department of Radiology Haaglanden Medical Center The Hague the Netherlands; ^10^ Department of Neurology Leiden University Medical Center Leiden the Netherlands; ^11^ Department of Neurology, University Hospital Bern, University of Bern Bern Switzerland; ^12^ Department of Radiology and Nuclear Medicine Maastricht University Medical Center Maastricht the Netherlands

**Keywords:** ischemic stroke, large vessel occlusion, medium vessel occlusion, thrombectomy, treatment time, Ischemic Stroke

## Abstract

**Background:**

In large vessel occlusion (LVO) stroke, shorter time from stroke onset to endovascular thrombectomy (EVT), and faster recanalization in the early window (<6 hours from last seen well) are associated with better outcomes. This association is less well known for patients with M2 occlusions.

**Methods:**

We conducted a pooled analysis of the MR CLEAN (Multicenter Randomized Clinical Trial of Endovascular Treatment of Acute Ischemic Stroke in the Netherlands) MED and NO‐IV trials and analyzed the effect of time delay in EVT‐treated LVO (grouped as intracranial internal carotid artery (ICA), ICA‐Terminus, or M1‐segment) and M2 occlusion patients with known stroke onset times. The primary outcome was the ordinal 90‐day modified Rankin Scale score. Secondary outcomes included 90‐day functional independence (modified Rankin Scale score 0–2) and mortality.

**Results:**

We included 94 patients with M2 occlusions and 423 with LVOs. In LVOs, longer times between stroke onset to start of EVT or to successful recanalization were associated with worse functional outcomes (adjusted common odds ratio [acOR], 0.92 [ 95% CI, 0.87–0.98] and acOR, 0.91 [95% CI, 0.86–0.95] per 15 minutes, respectively). Every 15‐minute delay to start of EVT reduced the probability of functional independence by 2.3%. For M2 occlusions, no significant association was found between time to EVT or successful recanalization and functional outcome (acOR, 1.09 [95% CI, 0.97–1.22] and acOR, 1.04 [95% CI, 0.92–1.19] per 15 minutes). Patients with LVOs showed a significant 0.7% increased probability of 90‐day mortality per 15‐minute delay in the start of EVT, whereas patients with M2 occlusions showed no significant association.

**Conclusions:**

Treatment delays worsen functional outcomes in patients with LVOs. However, this was not shown in patients with M2 occlusions, suggesting that outcomes after EVT for patients with M2 occlusions in the early window depend less on time to treatment.

**Nonstandard Abbreviations and Acronyms**
EVT: endovascular thrombectomy
LVO: large vessel occlusion
mRS: modified Rankin Scale
NIHSS: National Institutes of Health Stroke Scale

**Clinical Perspective**

**What Is New?**

- In contrast to acute ischemic stroke patients with a large vessel occlusion, we found no significant association between delays in endovascular treatment and clinical outcomes in patients with M2 occlusions.

**What Are the Clinical Implications?**

- Future studies should determine whether specific subgroups of patients with an M2 occlusion, based on imaging characteristics or collateral status, may still benefit from faster time to treatment.

In patients with an acute ischemic stroke due to a large vessel occlusion (LVO) in the anterior circulation, a shorter time from stroke onset to endovascular thrombectomy (EVT) in the early time window (<6 hour from last seen well) is associated with better outcomes.^1^, ^2^, ^3^ A meta‐analysis of 5 large clinical trials elucidated this strong temporal dependency, with treatment benefits becoming nonsignificant after 7.3 hours.^1^ Moreover, both the Dutch MR CLEAN (Multicenter Randomized Clinical Trial of Endovascular Treatment of Acute Ischemic Stroke in the Netherlands) registry and a large nationwide US registry demonstrated that earlier treatment correlates with higher rates of functional independence and lower mortality.^2^, ^3^

Although EVT is widely accepted for large vessel occlusions, its role in medium and distal vessel occlusions is less certain. Recent randomized controlled trials in acute ischemic stroke with medium vessel occlusions found that EVT did not improve functional outcomes or reduce mortality at 90 days compared with best medical management alone.^4^, ^5^ This raises the question whether time to treatment plays the same critical role as it does in LVOs. Prehospital recognition and response times may be prolonged in cases of M2 occlusions, because the neurological deficit is often less severe. Additionally, the in‐hospital detection of distal occlusions and the duration of the EVT procedure itself may be prolonged. The impact of delayed recanalization might be less severe in M2 occlusions due to the greater availability of collateral pathways compared with LVOs. This study aimed to compare the association of time to treatment with clinical outcome in patients with acute ischemic stroke with M2 occlusions and patients with LVOs in the early time window.

## METHODS

### Patient Population

We pooled data from the MR CLEAN (Multicenter Randomized Clinical Trial of Endovascular Treatment of Acute Ischemic Stroke in the Netherlands) MED trial and the MR CLEAN NO‐IV trial. These trials ran from January 2018 to January 2020^6^, ^7^ and included patients aged ≥18 years undergoing EVT because of acute ischemic stroke resulting from an occlusion in the anterior circulation (intracranial internal carotid artery (ICA), ICA‐Terminus, M1, or [proximal] M2). All patients had a neurological deficit of ≥2 on the National Institutes of Health Stroke Scale (NIHSS) and underwent EVT in the early window (<6 hours). The pooled trial population represents a typical EVT eligible patient population in the Netherlands. For clarity, EVT was not the study intervention in these trials. MR CLEAN MED studied the effect of periprocedural heparin or aspirin, whereas MR CLEAN NO‐IV studied the added benefit of intravenous thrombolytic treatment pre‐EVT. Exclusion criteria for each trial can be found in the respective study protocols.^8^, ^9^

For this study, we included only EVT‐treated patients with a known stroke onset time. To avoid confounding effects on clinical outcomes, we excluded patients who received additional heparin or aspirin in the MR CLEAN MED trial.

Ethical approval for the MR CLEAN MED and MR CLEAN NO‐IV trial was obtained from the central medical ethics committee and research board of the Erasmus MC University Medical Center, Rotterdam, the Netherlands (MEC 2017‐366 and MEC‐2017‐368, respectively). Deferred written informed consent was obtained for all patients. The data that support the findings of this study are available from the corresponding author upon reasonable request.

### Study Parameters

The primary outcome measure in this study was the ordinal modified Rankin Scale (mRS) score at 90 days, ranging from 0 (no disability) to 6 (death). Secondary outcomes included functional independence (mRS score of 0–2), mortality within 90 days, and NIHSS score at 24 hours.

Occlusion location was determined by an independent blinded core laboratory. We distinguished LVO (ICA, ICA‐T, and M1) and M2 occlusions, where an M2‐segment occlusion was defined as any occlusion of the middle cerebral artery (MCA) distal to the first bifurcation, excluding the anterior temporal branch of the MCA.

The time from stroke onset to EVT procedure was defined as the interval from stroke onset to groin puncture. The time from stroke onset to successful recanalization was defined as the interval from stroke onset to successful recanalization, assessed using the expanded Treatment in Cerebral Infarction score. Successful recanalization was specified as expanded Treatment in Cerebral Infarction score ≥2B. Only patients who achieved successful recanalization were included in the analysis of time from stroke onset to successful recanalization. However, all patients were included in the comparison of response times. For patients who did not successfully recanalize, we used the time from stroke onset to last contrast bolus for digital subtraction angiography. Prehospital response time was defined as the interval from stroke onset to arrival in the intervention center, including the door‐in‐door‐out time in the primary hospital and transfer to the intervention center for transfer patients. The in‐hospital response time was defined as the time interval from arrival in the intervention center to groin puncture. Finally, procedure time was defined as the time from groin puncture to successful recanalization or to last contrast bolus in patients who did not successfully recanalize.

### Missing Data

We used a multiple imputation by chained equation to handle missing data. The number of imputations was based on the fraction of missing information, using the R package *HowManyImputations* devised by Von Hippel.^10^

### Statistical Analysis

Patient baseline characteristics were analyzed in the crude data set. Differences in baseline variables between patients with LVOs and M2 occlusions were assessed using the Wilcoxon rank sum test, Pearson χ^2^ test, or Fisher exact test, where appropriate. Prehospital response time, in‐hospital response time, time from stroke onset to start of EVT, and time from stroke onset to successful recanalization were compared using the Wilcoxon rank sum test.

The associations between stroke onset to treatment and stroke onset to successful recanalization and mRS scores at 90 days, in patients with LVOs and in patients with M2 occlusions, were analyzed using ordinal logistic regression over the whole range of the mRS, in which mRS scores were inverted so that a higher odds represents better outcomes. Relevant covariates were determined using previous literature. The regression model was adjusted for age, sex, NIHSS score at baseline, and whether a patient was treated with intravenous thrombolysis. Common odds ratios and adjusted common odds ratios with 95% CIs were reported per 15 minutes increase in time.

To investigate whether the association between time to treatment and clinical outcome differed across occlusion groups, an interaction term between time to treatment and occlusion group was included in the model. Based on the results, stratified analyses were subsequently performed for each occlusion group. The proportional odds assumption was assessed using the Brant test. A sensitivity analysis excluding age as a confounder was also conducted.

The secondary outcomes, 90‐day functional independence, 90‐day mortality, and NIHSS at 24 hours, were analyzed using binary logistic regression or linear regression. These secondary outcomes were adjusted for the same covariates as the primary outcome. Odds ratios, adjusted odds ratio, unstandardized ß coefficients, and adjusted ß coefficients with 95% CIs were reported per 15 minutes increase in time.

All probabilities are calculated using the imputed data set and Rubin rules,^11^ with 95% CIs derived from between‐ and within‐imputation variance.

Finally, a nonlinear model using cubic splines was also used for analysis. The Akaike information criteria and Bayesian information criteria were used for model comparison.

All statistical tests were 2‐tailed. Statistical significance was set at *P<*0.05. Statistical analyses were conducted using RStudio (version 4.4.1).

## RESULTS

**Figure 1:**
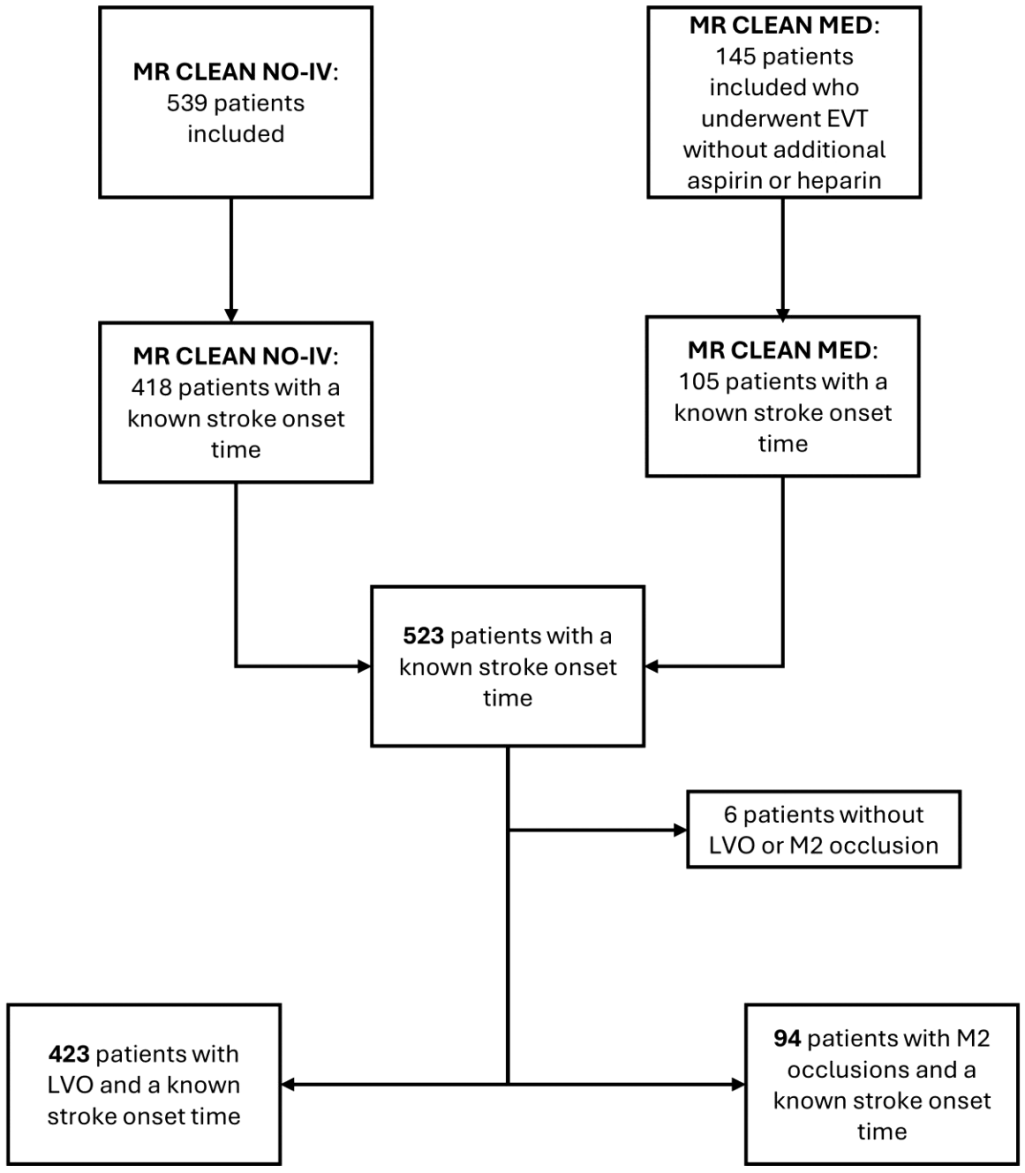
Flowchart of patient inclusion with EVT and LVO. EVT indicates endovascular thrombectomy; LVO, large vessel occlusion; and MR CLEAN, Multicenter Randomized Clinical Trial of Endovascular Treatment of Acute Ischemic Stroke in the Netherlands.

A total of 517 patients with known stroke onset time were included in this study. Of these, 423 patients had an LVO and 94 patients had an M2 occlusion (30 proximal, 55 distal, and 9 with unavailable imaging data to determine proximal or distal location). Subject count included in the analyses by trial were MR CLEAN NO‐IV (n=414) and MR CLEAN MED (n=103) (Figure 1). Baseline characteristics are described in Table 1.

**Table 1:** Baseline Characteristics Stratified by Occlusion Location

	LVO (n=423)	M2 (n=94)	*P* value
Age (y)	70±13	71±12	0.3
Sex, men	238 (56%)	56 (60%)	0.6
Medical history			
History of ischemic stroke	68 (16%)	19 (20%)	0.3
History of atrial fibrillation	54 (13%)	14 (15%)	0.6
History of diabetes	62 (15%)	24 (26%)	0.01
Prestroke mRS*			0.12
0: no symptoms	295 (70%)	73 (78%)	
1: minor symptoms, no limitations	80 (19%)	10 (11%)	
2: slight disability, no help needed	35 (8.3%)	5 (5.4%)	
mRS ≥3	13 (3.1%)	5 (5.4%)	
Baseline NIHSS	16 (11–20)	9 (7–14)	<0.001
Systolic blood pressure (mm Hg)†	149±24	154±23	0.051
ASPECTS	9 (8–10)	10 (9–10)	0.006
IVT administered	224 (53%)	50 (53%)	>0.9
Transferred from primary hospital	74 (17%)	24 (26%)	0.072
Time from stroke onset to door intervention center (min)‡	60 (45–120)	68 (45–130)	0.2
Time from door intervention center to groin puncture (min)§	59 (45–74)	62 (43–81)	0.3
Time from stroke onset to groin puncture (min)‖	129 (105–161)	145 (107–185)	0.03
Time from stroke onset to successful recanalization or last contrast bolus (min)¶	170 (141–196)	195 (167–228)	0.01
Procedure duration (min)#	46 (31–70)	50 (34–67)	0.8
Successful recanalization**	317 (82%)	63 (74%)	0.08
eTICI 2B	84 (22%)	15 (18%)	
eTICI 2C	51 (13%)	14 (16%)	
eTICI 3	182 (47%)	34 (40%)	

Data are presented as mean±SD, n (%), or median (interquartile range). ASPECTS indicates Alberta Stroke Program Early Computed Tomography Score; eTICI, expanded Treatment in Cerebral Infarction; IVT, intravenous thrombolysis; LVO, large vessel occlusion; mRS, modified Rankin Scale; and NIHSS, National Institutes of Health Stroke Scale.

* Data were missing for 1 patient (1 in M2).

^†^ Data were missing for 4 patients (3 in LVO, 1 in M2).

^‡^ Data were missing for 1 patient (1 in LVO).

^§^ Data were missing for 23 patients (20 in LVO, 3 in M2).

^‖^ Data were missing for 22 patients (19 in LVO, 3 in M2).

^¶^ Data were missing for 31 patients (25 in LVO, 6 in M2).

^#^ Data were missing for 28 patients (23 in LVO, 5 in M2).

** Data were missing for 47 patients (38 in LVO, 9 in M2).

There were no missing data in the outcome variables and 3.4% missing data in the relevant time metrics.

### Response Time and EVT Procedure

There was no statistically significant difference in prehospital response time or in‐hospital response time between patients with an LVO and patients with an M2 occlusion (Table 1). However, overall time from stroke onset to groin puncture and time from stroke onset to successful recanalization or last contrast bolus were significantly shorter for LVO compared with M2 occlusions (Table 1). Procedure duration was similar for LVO and M2 occlusions (Table 1). Successful recanalization rates were also similar for LVO and M2 occlusions. The majority of patients in both groups achieved an expanded Treatment in Cerebral Infarction 3 score, and the distribution of expanded Treatment in Cerebral Infarction scores was similar across the 2 occlusion types (Table 1).

### Time to Treatment and Outcomes

The effect of time to treatment on clinical outcome differed significantly between patients with LVOs and patients with M2 occlusions (*P* for interaction=0.006). Therefore, stratified analyses were performed to evaluate the association between time to treatment and outcome within each occlusion group separately.

Among individual predictors, only age (*P*<0.001) violated the proportional odds assumption, whereas all others satisfied it. The sensitivity analysis showed consistent direction and magnitude of the findings when age was excluded (Table S1). Despite the violation, age was retained in the model due to its well‐established role as a confounder in stroke research. Additionally, the proportional odds assumption was upheld for the outcome variable, supporting the model’s overall validity.

### Time to Treatment in LVO

For LVOs, a longer time from stroke onset to groin puncture was associated with a significant shift toward worse functional outcome at 90 days (per 15 minutes acOR, 0.92 [95% CI, 0.87–0.98]; Table 2). A prolonged time from stroke onset to successful recanalization was also associated with a significant shift toward worse functional outcome at 90 days (per 15 minutes acOR, 0.91 [95% CI, 0.86–0.95]; Table 2).

**Table 2:** Effect Estimates of Primary and Secondary Outcomes Per 15‐Minute Increase in Time

	Large vessel occlusions (n=423)		M2 occlusions (n=94)	
mRS ordinal	cOR [95% CI]	acOR* [95% CI]	cOR [95% CI]	acOR* [95% CI]
All patients	0.96 [0.92 to 1.02]	0.92 [0.87 to 0.98]	1.11 [0.99 to 1.24]	1.09 [0.97 to 1.22]
Recanalized patients	0.95 [0.90 to 1.01]	0.92 [0.86 to 0.98]	1.09 [0.96 to 1.23]	1.06 [0.93 to 1.21]
**Functional independence (mRS 0–2)**	**OR [95% CI]**	**aOR** * **[95% CI]**	**OR [95% CI]**	**aOR** * **[95% CI]**
All patients	0.94 [0.89 to 1.00]	0.91 [0.85 to 0.97]	1.10 [0.96 to 1.25]	1.07 [0.92 to 1.23]
Recanalized patients	0.93 [0.86 to 0.99]	0.89 [0.83 to 0.96]	1.07 [0.92 to 1.25]	1.02 [0.85 to 1.22]
**Mortality**	**OR [95% CI]**	**aOR** * **[95% CI]**	**OR [95% CI]**	**aOR** * **[95% CI]**
All patients	1.05 [0.98 to 1.13]	1.12 [1.02 to 1.22]	1.04 [0.86 to 1.25]	1.05 [0.86 to 1.29]
**24‐hour NIHSS**	**ß [95% CI]**	**Adjusted ß** * **[95% CI]**	**ß [95% CI]**	**Adjusted ß** * **[95% CI]**
All patients	0.32 [0.08 to 0.57]	0.60 [0.37 to 0.83]	−0.10 [−0.61 to 0.40]	0.05 [−0.41 to 0.50]
Recanalized patients	0.56 [0.34 to 0.79]	0.74 [0.55 to 0.93]	0.03 [−0.49 to 0.55]	0.21 [−0.28 to 0.71]

acOR indicates adjusted common odds ratio; cOR, common odds ratio; IVT, intravenous thrombolysis; NIHSS, National Institutes of Health Stroke Scale; and OR, odds ratio.

* Adjusted for age, sex, baseline NIHSS, and IVT.

**Figure 2:**
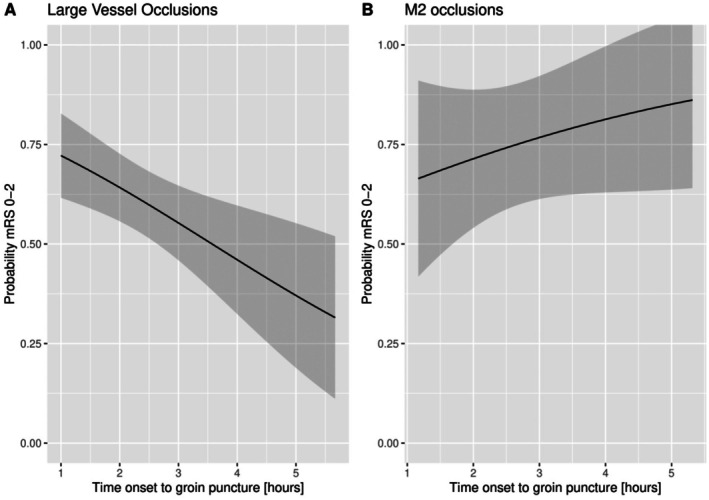
Probability distribution for functional independence (mRS 0–2) against time from stroke onset to groin puncture. Graphs were created using the fully adjusted models with the covariates fixed at their respective mean or mode. **A**, Probability distribution for large vessel occlusions in the early window. **B**, Probability distribution for M2 occlusions in the early window. mRS indicates modified Rankin Scale.

**Figure 3:**
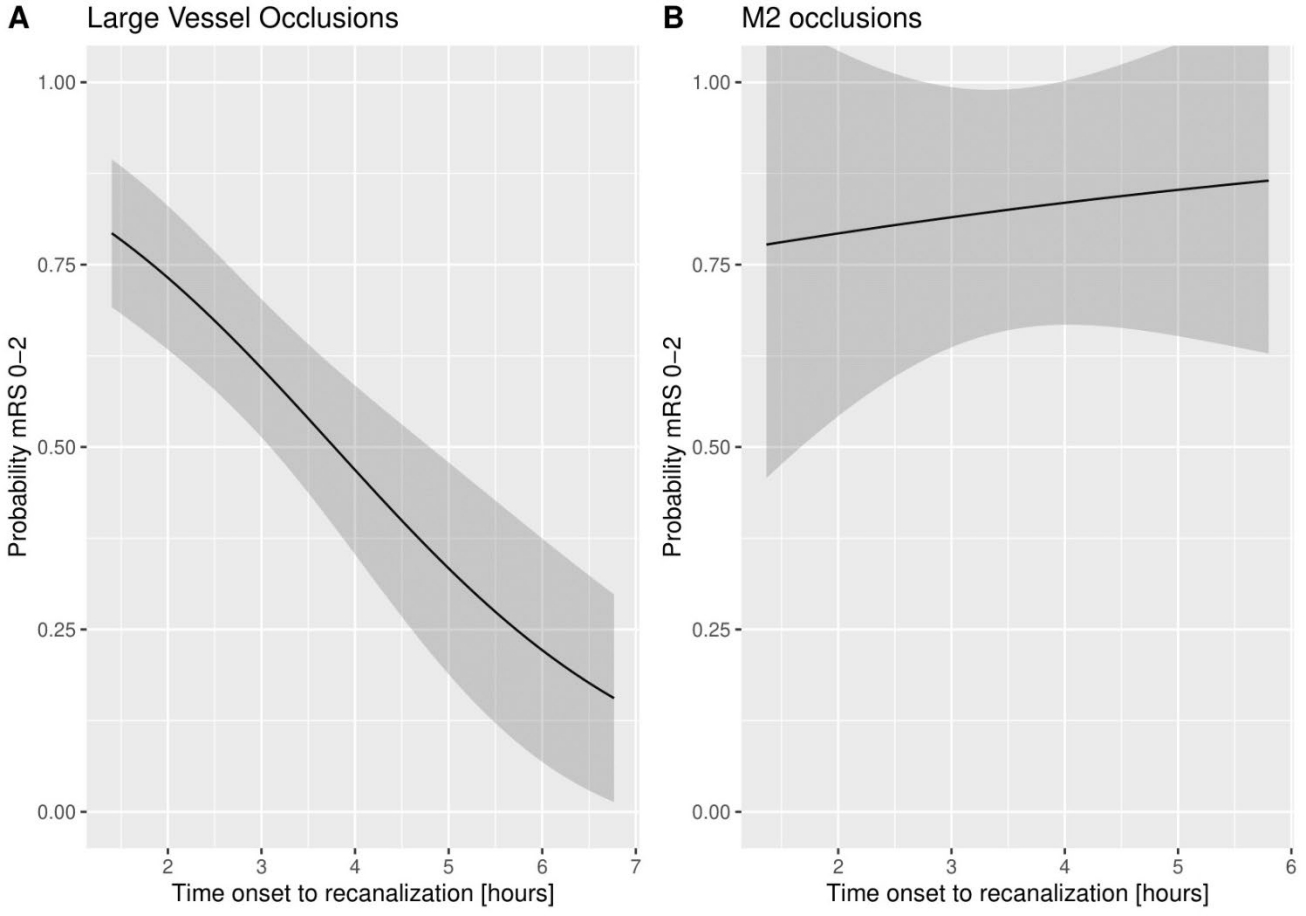
Probability distribution for functional independence (mRS 0–2) against time from stroke onset to successful recanalization. Graphs were created using the fully adjusted models with the covariates fixed at their respective mean or mode. **A**, Probability distribution for large vessel occlusions in the early window. **B**, Probability distribution for M2 occlusions in the early window. mRS indicates modified Rankin Scale.

In patients with an LVO, there was a significant negative correlation between time from stroke onset to groin puncture and functional independence (mRS score 0–2) at 90 days, as well as time from stroke onset to successful recanalization and functional independence (Figure 2A and Figure 3A). A 15‐minute delay in the start of the EVT procedure resulted in a 2.3% decrease in probability of functional independence (Table 2). Every 15‐minute delay from stroke onset to successful recanalization resulted in a 3.0% decrease in probability of functional independence (Table 2). Furthermore, the 24‐hour NIHSS score showed a significant increase of 0.62 and 0.75, respectively, for every 15‐minutes in delay to start of EVT and to successful recanalization (Table 2).

### Time to Treatment in M2

For M2 occlusions, there was no significant association between functional outcome and time from stroke onset to groin puncture or time from stroke onset to successful recanalization (per 15 minutes acOR, 1.09 [95% CI, 0.97–1.22] and acOR, 1.04 [95% CI, 0.92–1.19]; Table 2). Similarly, the 24‐hour NIHSS score did not show a significant association with time delay in either the start of EVT or successful recanalization (Table 2).

Furthermore, M2 occlusions showed no association between functional independence and time from stroke onset to groin puncture or time from stroke onset to successful recanalization (Figure 2B and Figure 3B).

Figures 2 and 3 indicate a potential nonlinear relationship. Consequently, a nonlinear model using cubic splines was also used for analysis. The Akaike information criterion and Bayesian information criterion were used for model comparison (Table S2). The results indicated that the linear model provided the best fit, and therefore this was used in all analysis.

### Mortality

**Figure 4:**
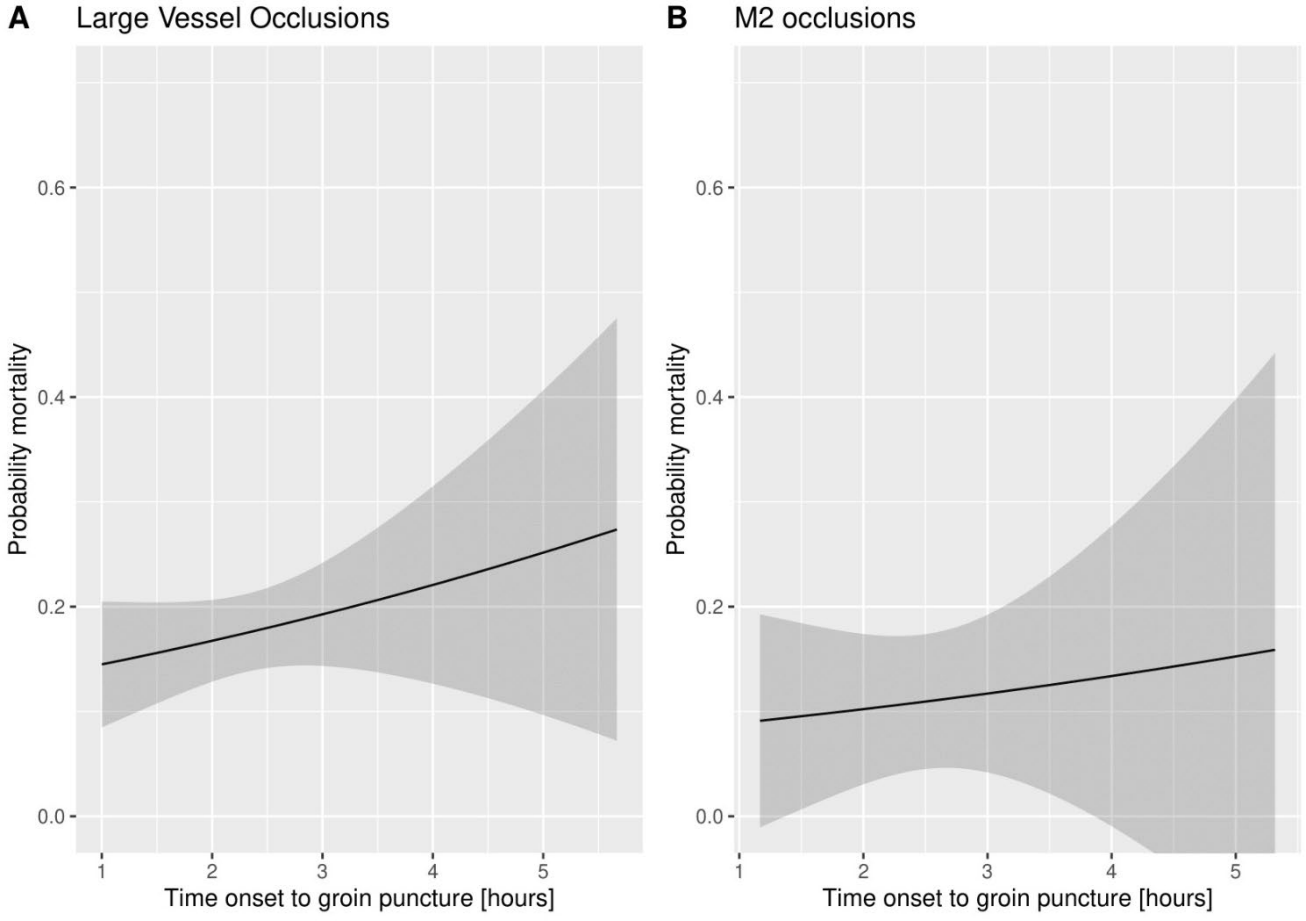
Probability distribution for mortality against time from stroke onset to groin puncture. **A**, Probability distribution for large vessel occlusions in the early window. **B**, Probability distribution for M2 occlusions in the early window.

In patients with an LVO, time from stroke onset to groin puncture was significantly associated with 90‐day mortality (Table 2). Every 15‐minute delay in the start of EVT resulted in a 0.7% increase in probability of mortality for patients with LVOs (Figure 4A). In contrast, in patients with M2 occlusions, time from stroke onset to groin puncture was not significantly associated with mortality. The probability distribution for mortality against time from stroke onset to groin puncture is shown in Figure 4B.

## DISCUSSION

In this study we investigated the association of time elapsed from stroke onset to start of EVT and successful recanalization with clinical outcome in patients with acute ischemic stroke in the early time window for patients with LVOs and M2 occlusions. We confirmed the previously established findings on the temporal effect of EVT in patients with LVOs; a longer time from stroke onset to start of EVT procedure or successful recanalization resulted in a decreased probability of functional independence. However, this association was not demonstrated in patients with M2 occlusions.

### Response Time and EVT Procedure

We found a significantly longer time from stroke onset to the start of EVT and to successful recanalization or last contrast bolus in patients with an M2 occlusion compared with patients with an LVO. This aligns with a previous study, which also reported longer stroke onset to successful recanalization intervals for M2 occlusions compared with M1 occlusions.^12^ The longer stroke onset to groin puncture time in M2 occlusions is attributable to the cumulative effect of delays across both prehospital and in‐hospital response time. These delays may be caused by patient delay due to milder deficits, prehospital stroke scales’ limited sensitivity in detecting more distal occlusions,^13^ and the greater difficulty of detecting M2 occlusions on CT angiography compared with LVO.^14^

### Large Vessel Occlusions

We found a 2.3% decrease in the probability of functional independence for every 15‐minute delay from stroke onset to the start of EVT, and a 3.0% decrease for every 15‐minute delay to successful recanalization for LVOs. These values are higher compared with the literature (1.3% and 1.9%, respectively^3^). However, prior studies reflect different patient selection, procedural efficacy, and safety standards. Furthermore, although predominantly LVO, the previous study included a small number of M2 occlusions (12%), which may have flattened the overall time‐dependent outcome curve, thereby misrepresenting the functional independence probabilities specific to LVOs.

### M2 Occlusions

For patients with an M2 occlusion, no significant association was found between time from stroke onset to treatment and functional outcome. Recent randomized controlled trials on EVT in M2 and more distal occlusions found no overall benefit of EVT over best medical treatment.^4^, ^5^ However, these studies mainly included co‐/nondominant M2 occlusions and more distal M2/M3 occlusions. In contrast, our study included both dominant and co‐/nondominant M2 occlusions, with a predominance of proximal M2 occlusions. Previous studies suggested a benefit of EVT for dominant/proximal M2 occlusions.^15^

The absence of an association between time to treatment and outcomes in our study could be explained by the limited sample size, possibly leading to a type II error. However, the point estimate suggests a much smaller effect compared with LVOs. This might be due to the better collateral circulation in M2 occlusions compared with LVOs. There are more opportunities for blood supply via the secondary collaterals, including the leptomeningeal vessels, in M2 occlusions.^16^, ^17^ For LVOs, the collateral circulation mainly relies on the primary collaterals, composed of the circle of Willis,^16^ with limited leptomeningeal collateral flow due to the reduced perfused brain tissue around the occlusion.^17^ This is further supported by a study that found that time dependency of outcomes after EVT is more pronounced when parts of the proximal MCA territory, which has less collateralization, are affected.^18^ Similarly, another study found that patients with milder baseline deficits and better collaterals are less affected by treatment delays,^19^ further supporting the idea that collaterals play a key role in mitigating time dependency. Furthermore, LVOs affect a larger area, where the collateral circulation may be insufficient to compensate for the reduced blood flow, whereas M2 occlusions affect a smaller area where the collateral circulation is able to sustain adequate perfusion. Another explanation for the absence of a clear correlation between time to treatment and functional outcome may lie in the generally less severe outcomes in patients with M2 occlusions. It is conceivable that the mRS does not accurately reflect outcome differences for M2 occlusions, and a time effect may still be present, although our secondary analysis using a 24‐hour NIHSS score did not show a time effect either.

A strength of this study is that it uses data from 2 randomized controlled trials with limited missing data, as well as protocoled and blinded outcome measurement. Furthermore, there were no strict inclusion and exclusion criteria for EVT in these trials, which makes the patient population representative for general practice.

A limitation of this study is that the majority of the included M2 occlusions are proximal M2 occlusions, because only proximal M2 occlusions were eligible in the MR CLEAN MED and NO‐IV trials. However, several distal M2 occlusions were inadvertently included. Consequently, our results may not adequately reflect the temporal effects in true distal M2 occlusions. Another limitation of this study is that it only included strokes with a known stroke onset time, which reduced the sample size and precluded adjustments for covariates in the ordinal regression analysis. Furthermore, this study did not investigate the temporal effects in the late window due to the limited number of strokes with a known stroke onset time in this window. In cases with unwitnessed stroke onset, the most accurate proxy for stroke onset remains uncertain. As a result, the complete picture of the temporal effects in M2 occlusions remains unclear, and further research is needed. Finally, although we know from intravenous thrombolysis studies in stroke that the first golden hour is crucial for increasing good outcome,^20^ no patients with M2 occlusions underwent EVT within an hour after stroke onset. Therefore, we cannot rule out the possibility that we may have missed an ultraearly time‐dependent effect of EVT.

## CONCLUSIONS

Patients with an LVO ischemic stroke demonstrate a negative correlation between time to treatment and outcome, where delays in EVT treatment are associated with poorer clinical outcomes. However, this correlation is not found in patients with M2 occlusions. This suggests that outcomes after EVT for patients with M2 occlusions in the early window depend less on time to treatment. Further research in larger data sets, extending outside the early window, is needed to gain more insight in time‐to‐treatment effects on clinical outcomes after EVT in patients with M2 occlusions.

## Sources of Funding

The MR CLEAN MED and NO‐IV trials were part of the CONTRAST (Collaboration for New Treatments of Acute Stroke) consortium. The CONTRAST consortium acknowledges the support from the Netherlands Cardiovascular Research Initiative, an initiative of the Dutch Heart Foundation (CVON2015‐01: CONTRAST), and from the Brain Foundation Netherlands (HA2015.01.06). The CONTRAST consortium is additionally financed by the Ministry of Economic Affairs by means of the Public‐Private Partnership (PPP) Allowance made available by Top Sector Life Sciences and Health to stimulate public–private partnerships (LSHM17016). In addition, the CONTRAST consortium was funded in part through unrestricted funding by Stryker, Medtronic, and Penumbra. The funding sources were not involved in study design, monitoring, data collection, statistical analyses, interpretation of results, or article writing.

## Disclosures

J.S. reports speaker fees from Medtronic. W.v.Z. reports consulting fees from Philips and speaker fees from Stryker, Cerenovus, and Nicolab (all paid to institution). Moreover, he is a participant in the Safety Monitoring Board or Advisory Board of the WE‐TRUST (Philips), IN EXTREMIS (Centre Hospitalier Universitaire Montpellier), ANAIS (Anaconda), and TECNO (University Hospital Basel) studies. U.F. reports research support of the Swiss National Science Foundation and the Swiss Heart Foundation; principal investigator of the ELAN trial; co‐principal investigator of the DISTAL, TECNO, SWIFT DIRECT, SWITCH, ELAPSE, and ICARUS trials; research grants from Medtronic (BEYOND SWIFT, SWIFT DIRECT) and from Stryker, Rapid Medical, Penumbra, Medtronic, Phenox (DISTAL), and Boehringer Ingelheim (TECNO); consultancies for Medtronic (fees paid to institution); participation in an advisory board for AstraZeneca (former Alexion/Portola), Bayer, Boehringer Ingelheim, Biogen, AbbVie, Siemens (fees paid to institution); member of a clinical event committee of the COATING study (Phenox); member of the data and safety monitoring committee of the TITAN, LATE_MT, IN EXTREMIS, and RapidPulse trials; president of the Swiss Neurological Society; and president‐elect of the European Stroke Organization. C.M. reports research grants from Stryker, Boehringer Ingelheim (paid to institution) and is a (minority interest) shareholder of Nicolab. B.R. reports research support of the Dutch Heart Foundation and the Netherlands Organization for the Health Research and Development (ZonMw) and speaker fees from Medtronic, all paid to the institution. The remaining authors have no disclosures to report.

## Supporting information

Tables S1–S2
