# Supplementary material for: Time to Endovascular Treatment and Clinical Outcome in Acute Ischemic Stroke With M2‐Segment Occlusion
Source: J Am Heart Assoc. 2025 Nov 11;14(22):e043984. doi: 10.1161/JAHA.125.043984 (PMC12887208; doi:10.1161/JAHA.125.043984)
Supplement: Supplementary file 1 — Tables S1–S2 [file JAH3-14-e043984-s001.pdf]

Supplemental Material

Table S1: Sensitivity analysis – adjusted effect estimates of primary and secondary outcomes per 15 minute increase in time with age not included as confounder.

	Large Vessel Occlusions (n=423)	M2 occlusions (n=94)
	**acOR* [95% CI]**	**acOR* [95% CI]**
**mRS ordinal**		
All patients	0.91 [0.86 to 0.96]	1.09 [0.97 to 1.22]
Recanalized patients	0.91 [0.86 to 0.97]	1.06 [0.93 to 1.21]
	**aOR* [95% CI]**	**aOR* [95% CI]**
**Functional independence (mRS 0-2)**		
All patients	0.90 [0.84 to 0.96]	1.07 [0.92 to 1.23]

Recanalized patients	0.89 [0.83 to 0.96]	1.02 [0.85 to 1.22]
**Mortality**		
All patients	1.12 [1.04 to 1.22]	1.06 [0.86 to 1.30]

	aß* **[95% CI]**	aß* **[95% CI]**
**24h NIHSS**		
All patients	0.62 [0.39 to 0.85]	0.05 [-0.40 to 0.51]
Recanalized patients	0.75 [0.54 to 0.96]	0.21 [-0.28 to 0.70]

* Adjusted for sex, baseline NIHSS and IVT given with NIHSS: National Institutes of Health Stroke Scale and IVT: intravenous thrombolysis.

Table S2: Akaike Information Criterion and Bayesian Information Criterion for different models

	AIC-value	BIC-value
**Large Vessel Occlusion**		
Linear model	525.68	549.97
Non-linear model (Cubic splines*)	1276.49	1308.87
**M2 occlusion**		
Linear model	118.08	133.34
Non-linear model (Cubic splines*)	157.73	178.07

*Cubic splines model defined using 3 degrees of freedom
